# Multidrug Resistance Profiles and Resistance Mechanisms to β-Lactams and Fluoroquinolones in Bacterial Isolates from Hospital Wastewater in Bangladesh

**DOI:** 10.3390/cimb45080409

**Published:** 2023-08-05

**Authors:** Rasel Khan Manik, Zimam Mahmud, Israt Dilruba Mishu, Md Sourav Hossen, Zakir Hossain Howlader, A. H. M. Nurun Nabi

**Affiliations:** 1 Department of Biochemistry and Molecular Biology, University of Dhaka, Dhaka 1000, Bangladesh; 2 Department of Microbiology, University of Dhaka, Dhaka 1000, Bangladesh; 3 Laboratory of Population Genetics, Department of Biochemistry and Molecular Biology, University of Dhaka, Dhaka 1000, Bangladesh

**Keywords:** multidrug resistance, β-lactam antibiotics, quinolone resistance, QRDR mutations

## Abstract

Multidrug resistance (MDR) is one of the deadliest public health concerns of the 21st century, rendering many powerful antibiotics ineffective. The current study provides important insights into the prevalence and mechanisms of antibiotic resistance in hospital wastewater isolates. In this study, we determined the MDR profile of 68 bacterial isolates collected from five different hospitals in Dhaka, Bangladesh. Of them, 48 bacterial isolates were identified as *Enterobacteriaceae*. Additionally, we investigated the prevalence and distribution of five beta-lactam resistance genes, as well as quinolone resistance mechanisms among the isolates. The results of this study showed that 87% of the wastewater isolates were resistant to at least three different antibiotic classes, as revealed using the disc diffusion method. Resistance to β-lactams was the most common, with 88.24% of the isolates being resistant, closely followed by macrolides (80.88% resistant). Polymyxin was found to be the most effective against wastewater isolates, with 29.41% resistant isolates. The most common β-lactam resistance genes found in wastewater isolates were *bla_TEM_* (76.09%), *bla_CTX-M1_* (71.74%), and *bla_NDM_* (67.39%). Two missense mutations in the quinolone resistance-determining region (QRDR) of *gyrA* (S83L and D87N) and one in both *parC* (S80I) and *parE* (S458A) were identified in all isolates, and one in *parE* (I529L), which had not previously been identified in Bangladesh. These findings suggest that hospital wastewater acts as an important reservoir of antibiotic-resistant bacteria wherein resistance mechanisms to β-lactams and fluoroquinolones are obvious. Our data also emphasize the need for establishing a nationwide surveillance system for antibiotic resistance monitoring to ensure that hospitals sanitize their wastewater before disposal, and regulation to ensure hospital wastewater is kept away from community settings.

## 1. Introduction

Antimicrobial resistance (AMR) is a global health issue that is of growing concern to researchers and medical professionals worldwide. The growth and spread of bacteria that are resistant to multiple classes of antibiotics pose a serious risk to human health, increasing mortality, morbidity, and medical expenses. Multidrug resistance frequently results in complete treatment failure, and spreads virulence factors in both healthcare facilities and community settings. Moreover, antibiotic resistance has far-reaching economic and societal impacts, including reduced productivity, impaired economic growth, and increased poverty [1]. Therefore, there is an urgent need to understand the prevalence and distribution of antibiotic resistance genes to shed light on the development of effective treatment strategies and infection control measures.

The mechanisms of resistance involve a variety of strategies, such as upregulating the genes of efflux proteins [2], downregulating the genes of influx proteins [2], acquiring antibiotic-resistant genes via horizontal gene transfer from already resistant bacteria [3,4], and mutating antibiotic resistance genes such as *gyrA, gyrB, parC, parE,* etc. [5]. These resistance genes are frequently encoded on plasmids or other mobile genetic elements, thus facilitating swift transfer both within and among bacterial populations [6].

Beta-lactam antibiotics are among the most abundantly prescribed antimicrobials, and the rising prevalence of beta-lactam resistance genes in bacterial populations compromises their effectiveness. Beta-lactam resistance genes are more likely to arise and propagate in bacterial populations as a result of the extensive usage of beta-lactam antibiotics, improper disposal of hospital wastes, and unprescribed extensive uses in livestock farming [7]. Beta-lactam resistance is generally caused by enzymes that break down the antibiotic’s beta-lactam ring, rendering it ineffective against bacteria. Genes for these enzymes, such as *bla_TEM_*, *bla_SHV1_*, *bla_CTX-M1_*, *bla_NDM_*, *bla_VIM1_*, etc., have been identified in several Gram-negative bacterial species, with particularly higher prevalence observed in *Escherichia coli* [8,9].

Quinolone resistance, on the other hand, is brought on by mutations in the *gyrA*, *gyrB*, *parC*, and *parE* genes, which lead to a reduction in drug-binding affinity [10,11,12]. These genes are expressed constitutively and require additional mutations in quinolone resistance-determining regions [12]. Ciprofloxacin, levofloxacin, nalidixic acid, moxifloxacin, and other commonly prescribed quinolones lose their efficacy when the *gyrA, gyrB, parC,* and *parE* genes are mutated, resulting in altered quinolone binding or decreased affinity of the protein products to quinolones [10,13,14]. Critical missense mutations in particular regions of these gyrase and topoisomerase IV genes have a significant impact on the ability of fluoroquinolone drugs to form optimal conformation and affinity with their targeted proteins [14,15]. These regions, which contain missense mutations resulting in such effects, are termed quinolone resistance-determining regions (QRDRs).

Antibiotic resistance is even more concerning, because resistance to one class of antibiotics due to their overuse and unsafe disposal encourages the development of resistance to other antibiotic classes [2,16]. The prevalence of ESBL-producing *Klebsiella* spp., *E. coli*, and carbapenem-resistant *Enterobacteriaceae*, for example, was positively linked with increased carbapenem and polymyxin intake [17]. Simultaneously, a declining tendency in the innovation of new antibiotics has been observed over the years due to the inadequate profitability of long-term medicinal products, regulatory constraints, and technical complexities.

A combination of monitoring and surveillance to prevent the spread of antibiotic-resistant bacteria and their sources and application of jurisdiction has been found to be effective in combatting antibiotic resistance [18,19]. Our study investigates the prevalence of resistance in different species of bacteria isolates from hospital wastewater, and, therefore underscores the importance of monitoring antibiotic resistance in clinical settings and provides valuable insight into the prevalence and distribution of beta-lactam resistance genes among bacterial isolates from different hospital settings. Additionally, our efforts to detect mutations and their effects that cause fluoroquinolone resistance present a mutational spectrum of hospital wastewater bacteria. Our research also provides a foundation for future studies on the mechanisms and distribution of antibiotic resistance genes in Bangladesh, and contributes to the global effort to combat antibiotic resistance in clinical settings.

## 2. Methods and Materials

### 2.1. Selection of Sampling Sites

**Figure 1:**
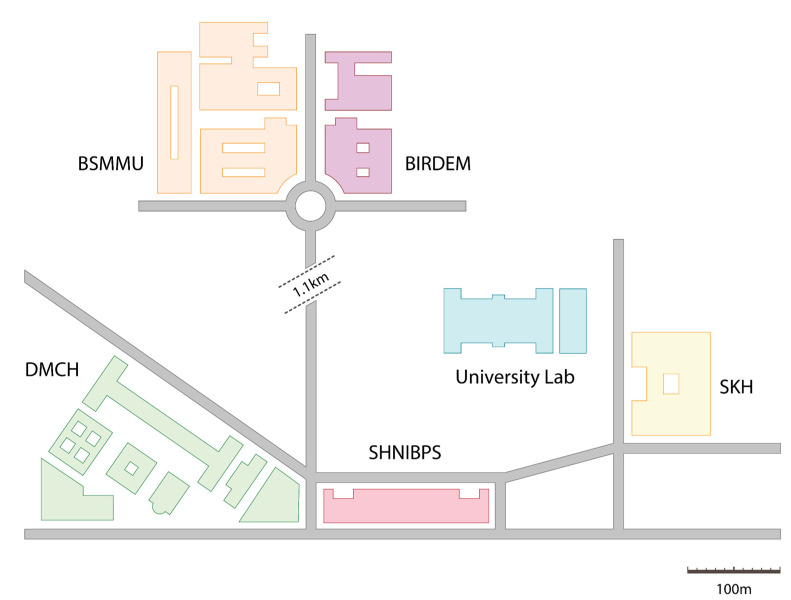
Map of the study area.

The area of research was inclusive of five medical centers present within a distance of one kilometer from the Biochemistry and Molecular Biology Laboratory situated at the highly regarded University of Dhaka. It is from this location that the wet experiments and analyses were conducted, as illustrated in Figure 1. These hospitals have unencumbered access to the municipal sewage drains, and as a result are capable of transporting waste and microbiome to the surrounding community settings. These hospitals include the Bangladesh Institute of Research and Rehabilitation in Diabetes, Endocrine, and Metabolic Disorders (BIRDEM), Bangabandhu Sheikh Mujib Medical University (BSMMU), Dhaka Medical College and Hospital (DMCH), Sheikh Hasina National Institute of Burn and Plastic Surgery (SHNIBPS), and Sarkari Karmachari Hospital (SKH).

### 2.2. Sample Collection

**Figure 2:**
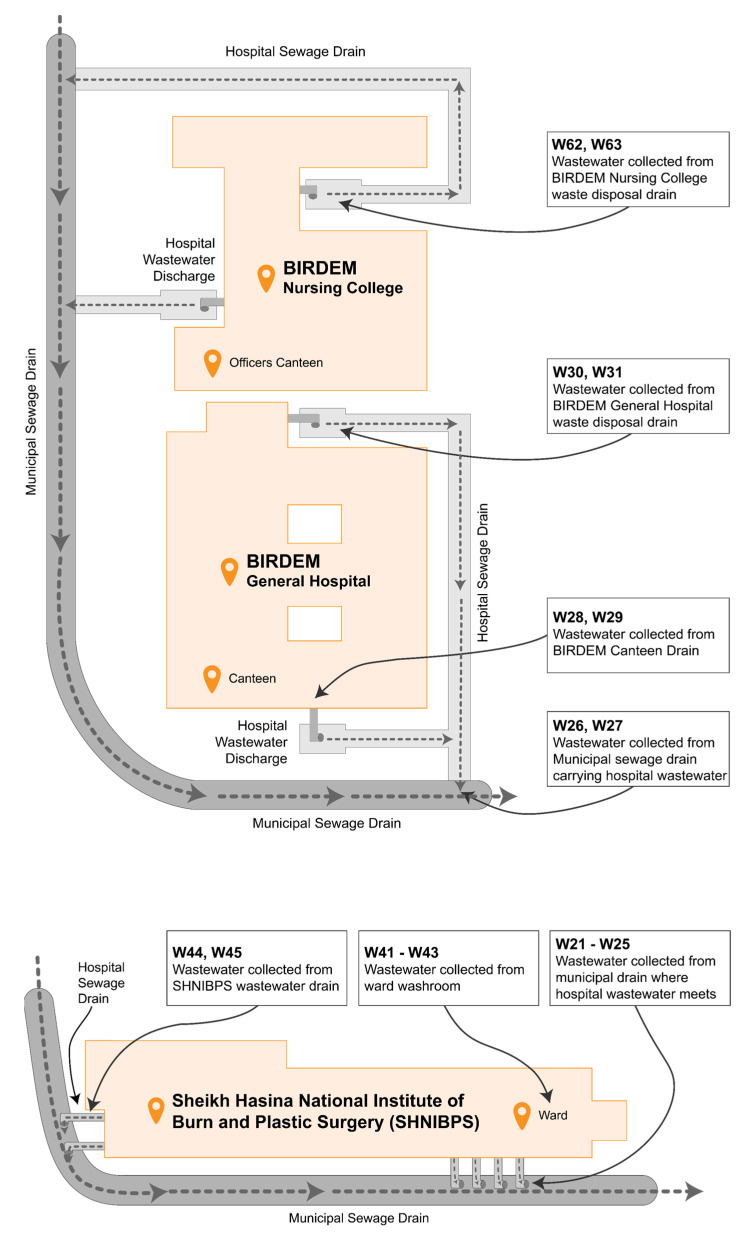
Map of sample collection sites in BIRDEM and SHNIBPS.

A total of 68 bacterial isolates were collected from five different hospital settings in Dhaka, Bangladesh, primarily from but not limited to hospital wastewater drains. Other sources of samples include hospital washroom water, rainwater accumulated alongside waste disposal sites, etc. All the included wastewater discharges have access to municipal sewage drains. Figure 2 depicts the site map pertaining to the sample collection process from BIRDEM and SHNIBPS, while simultaneously elucidating how these wastewater channels attain entry into the municipal drainage system.

From each of the sample collection sites, 5.0 mL of wastewater was collected in falcon tubes and temporarily stored in an icebox, transported into the laboratory, filtered to remove organic and inorganic pollutants, and diluted to 100× and 1000× before inoculating in differential media. The specimens underwent preparation and identification processes while adhering to the mandatory safety precautions within a Biosafety Level 2 (BSL2) laboratory.

### 2.3. Selective Media and Bacterial Identification

We wanted to investigate resistance, particularly among Gram-negative bacteria from hospital wastewater sources; we selected four Gram-negative isolates and one Gram-positive isolate. The team allowed the wastewater bacterial population to grow in four selective media to isolate five distinct bacterial species. *Escherichia coli* and *Klebsiella* spp. isolates were identified using MacConkey Agar media, while *Staphylococcus aureus*, *Vibrio cholerae*, and *Pseudomonas aeruginosa* isolates were selectively grown in Mannitol Salt Agar, TCBS Agar, and Cetrimide Agar media, respectively. These selective media possess exceedingly stringent specifications to screen exclusively for specific strains. Each one of the isolates was sub-cultured three times to eliminate any false-positive identification and to obtain a pure culture of the bacteria of interest. These media along with Mueller Hinton Broth (MHB) and Mueller Hinton Agar (MHA) media were purchased from HiMedia Laboratories Pvt Ltd., Maharashtra, India. Some 500 mL of pure culture broth was added to an equal amount of 50% glycerol, and stored at −80 °C for longer shelf life.

### 2.4. Antimicrobial Susceptibility Test

Bacterial sensitivity towards each of the antibiotics was evaluated following the Kirby–Bauer disc diffusion protocol, and the zone of inhibition (ZOI) was interpreted following the Clinical and Laboratory Standards Institute (CLSI-2022) and European Committee on Antimicrobial Susceptibility Testing (EUCAST-2022) guidelines. Isolates were incubated in MHB for two hours at 37 °C, and microbial growth was adjusted to the 0.5 McFarland standard before preparing the lawn on MHA plates, placing antibiotic discs, and incubating overnight at 37 °C.

A total of 11 different antibiotic discs of 7 antibiotic classes were purchased from Oxoid Ltd., Basingstoke, UK. The list and classes of antibiotics are presented in Table 1.

### 2.5. DNA Extraction and Resistance Gene Screening

The genomic DNA of bacteria was extracted through a modified boiling DNA extraction protocol [20,21], which consisted of a sequence of centrifugations, boiling at 100 °C, and cooling to 0 °C. Initially, a 1mL sample of liquid culture grown overnight was centrifuged at 10,000 rpm for 10 min, and the supernatant was subsequently discarded. The resulting pellet was rinsed with 0.9% saline solution and centrifuged once more, following which 300 µL of ddH_2_O was added to dissolve the pellet. The solution was then boiled at 100 °C and immediately placed on ice to cool to 0 °C. The chilled solution was at last subjected to centrifugation at 10,000 rpm, and 100 µL of the supernatant was amassed and preserved at a temperature of −20 °C.

The prevalence of five extended spectrum beta-lactamase (ESBL) and four fluoroquinolone resistance genes was investigated using polymerase chain reaction (PCR) and visualized using subsequent gel electrophoresis. Primer sequences for ESBL resistance-determining genes *(bla_NDM_*, *bla_CTX-M1_*, *bla_TEM_*, *bla_SHV1_*, *bla_VIM1_)*, as well as the quinolone resistance-determining region (QRDR) of DNA gyrase and topoisomerase genes *(gyrA*, *gyrB*, *parC*, *parE),* were selected from earlier studies and synthesized by Macrogen, Inc. The specifications of the primers used in the current study, which target and amplify particular regions of the relevant genes in the genomes of the bacterial isolates, are listed in Table 2. 

A negative control was included in each batch of PCR. PCR amplicons were resolved in 1.0% agarose gel at 75 V for 60 min and visualized in the AlphaImager Mini HP Gel documentation system (model MLB-26C, manufactured by Cell Biosinces Inc., Taiwan).

### 2.6. DNA Sequencing and Mutation Analysis

QRDRs of DNA gyrase and topoisomerase genes (*gyrA, gyrB, and parC, parE*) of four isolates were sequenced through BTSeq™ Contiguous Sequencing by Celemics, Inc., Geumcheon-gu, Seoul, Korea. Mutational analysis was performed in Geneious Prime software (version 2023.0.1, build 2022-11-28 12:49). DNA sequences of the target regions of all four genes were aligned with the annotated reference gene sequence of *Escherichia coli* (INSCD-U00096) retrieved from the NCBI Genome Browser (accessed on 19/02/2023), and an appropriate reading frame was selected to avoid an internal stop codon. Any mismatch with the reference sequence was labeled as a point mutation, and any point mutation that resulted in a changed amino acid sequence was designated as a missense mutation, which was later examined to identify its significance in drug–protein interactions.

### 2.7. Molecular Docking Analysis

The wild-type protein structures of *gyrA*, *parC*, and *parE* were obtained from RCSB Protein Data Bank (accessed on 17 March 2023) [27], and the homology-based 3D structure was predicted using SWISS-MODEL (accessed on 20 March 2023) [28]. The optimal binding site on ciprofloxacin, the position of the protein–antibiotic interaction, and the affinity for ciprofloxacin of both wild-type and mutated proteins were predicted using AutoDock4 (version 4.2.6) [29], following the method described by Mahmud et al. [30].

### 2.8. Statistical Analysis

Statistical analysis was performed using Microsoft Excel 2021 (version 2212) and GraphPad Prism (version 9.5.0.730). GraphPad Prism was used to generate graphs and figures.

## 3. Results

### 3.1. Antibiotic Resistance Profile of Bacteria Isolated from Different Hospital Settings

**Figure 3:**
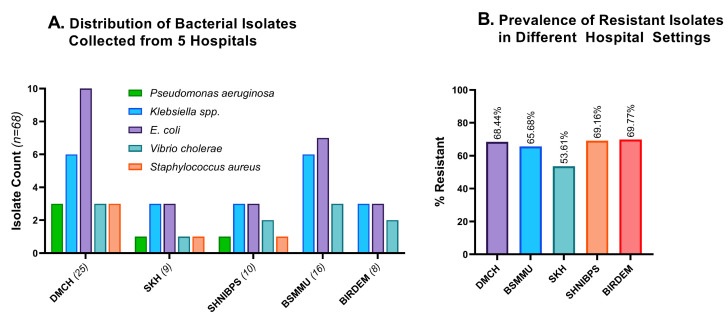
Distribution and resistance profiles of different hospitals’ wastewater. (**A**). Distribution of 68 bacterial isolates grouped according to the source of wastewater. The majority of the isolates were collected from DMCH, then from BSMMU and SHNIBPS. (**B**). Comparative resistance profiles of the isolates from five hospital settings. BIRDEM, BIRDEM General Hospital; DMCH, Dhaka Medical College and Hospital; BSMMU, Bangabandhu Sheikh Mujib Medical University; SHNIBPS, Sheikh Hasina National Institute of Burn and Plastic Surgery; SKH, Sarkari Karmachari Hospital.

Figure 3A indicates the demography of the 68 bacterial isolates that we obtained from five hospital sites. Some 36.8% of the bacterial isolates were collected from the DMCH, 23.5% from BSMMU, 14.7% from SHNIBPS, 12.2% from SKH, and 11.8% from BIRDEM General Hospital.

Excessive application and unsafe disposal of medical wastes play a significant role in inducing resistance to diverse species of bacteria [31]. Therefore, hospitals with larger patient populations experience an increase in antimicrobial resistance. All the experimental hospital areas harbored a significant number of resistant isolates, with BIRDEM and SHNIBPS having the highest resistance rate (>69%). The number of resistant isolates in the other three hospital areas was also alarmingly high, with DMCH, BSMMU, and SKH carrying 68.44%, 65.68%, and 53.61% resistant isolates, respectively (Figure 3B). SKH has significantly fewer incidences of antimicrobial resistance compared to the average of the other four hospital areas (*p =* 0.0425).

### 3.2. Resistance against Commercially Available Antibiotics

**Figure 4:**
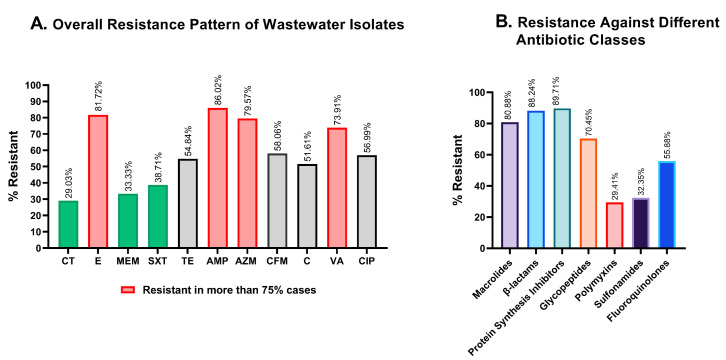
Graphical representation of the resistance profiles of hospital wastewater isolates. (**A**). Overall antimicrobial resistance against each of the antibiotics tested. Colistin and meropenem showed great effectiveness in killing wastewater bacteria, while erythromycin, ampicillin, and azithromycin were the least active against wastewater isolates. (**B**). Comparative resistance profiles of the isolates against seven different antibiotic classes. CT: colistin; E: erythromycin; MEM: meropenem; SXT: sulfamethoxazole-trimethoprim; TE: tetracycline; AMP: ampicillin, AZM: azithromycin; CFM: cefixime; C: chloramphenicol; VA: vancomycin; CIP: ciprofloxacin.

More than 75% of the isolates in wastewater isolates showed resistance against ampicillin (86.02%), erythromycin (81.72%), azithromycin (79.57%), and vancomycin (73.91%) (Figure 4A, pink), while colistin, meropenem, and sulfamethoxazole-trimethoprim were the most potent against wastewater bacteria, with 29.03%, 33.33%, and 38.71% of isolates being resistant, respectively (Figure 4A, green). Compared to the four least effective antimicrobials, resistance to chloramphenicol, tetracycline, cefixime, and ciprofloxacin was less widespread, but still concerning with 51.61%, 54.84%, 58.06%, and 56.99% resistant isolates, respectively (Figure 4A, gray).

### 3.3. Resistance against Different Classes of Antibiotics

To determine resistance against antibiotic classes, the non-sensitivity of at least one of the antibiotics was taken into consideration for classes with multiple antibiotics. Polymyxin and sulfonamide showed a substantially better response in microbial suppression, with 29.41% and 32.35% inefficiency, while macrolides, β-lactams, and protein synthesis inhibitors were found to be ineffective in 80.88%, 88.24%, and 89.71% of the isolates, respectively (Figure 4B).

### 3.4. Resistance Profile of Different Bacterial Species

We exposed each of the isolates to all 11 antimicrobials to evaluate their responses. *E. coli*, *Klebsiella* spp., and *Vibrio cholerae* together made up 85% of the isolates, and the resistance patterns of these species against different antibiotics varied significantly. *E. coli* and *Klebsiella* spp. were observed to be highly resistant to erythromycin (96.08% and 90.48%), azithromycin (90.02% and 90.48%), ampicillin (82.35% and 90.48%), ciprofloxacin (62.75% and 66.67%), and tetracycline (56.86% and 71.43%), whereas *Vibrio cholerae* were almost absolutely resistant to colistin (100%), ampicillin (100%), cefixime (100%), chloramphenicol (100%), and meropenem (90.91%). According to this study, polymyxin was most effective against *E. coli* and *Klebsiella* spp., while sulfonamide was most effective against *Vibrio cholerae*. Although colistin and meropenem were extremely effective against *E. coli* and *Klebsiella* spp., they were completely ineffective in inhibiting *Vibrio cholerae* (Table 3).

### 3.5. β-Lactam Resistance Profile

**Figure 5:**
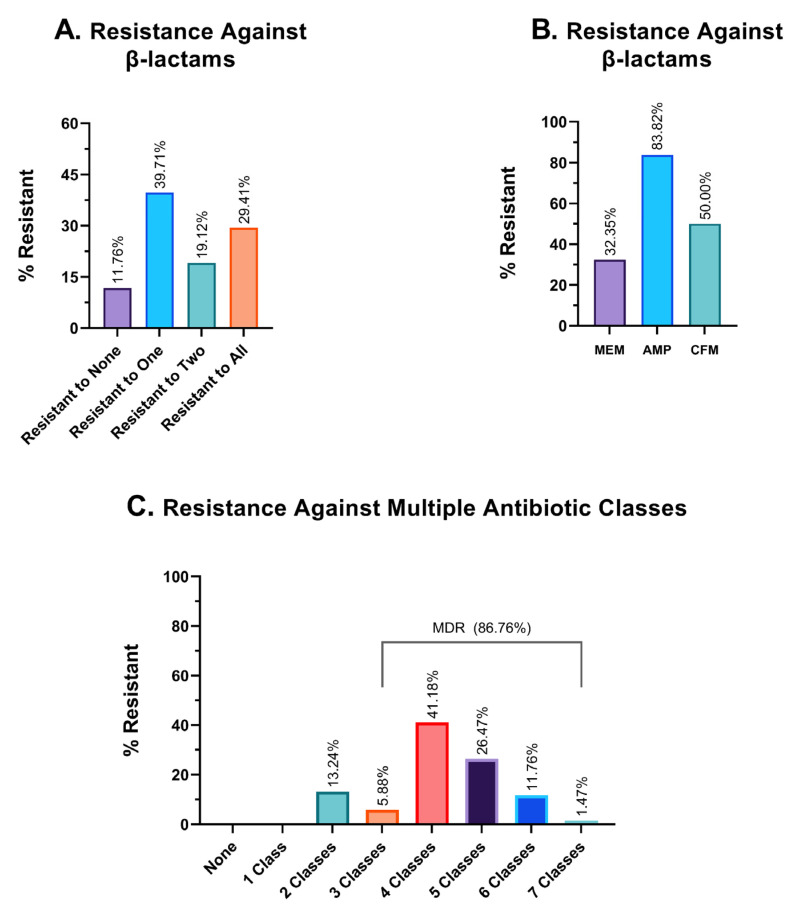
Graphical representation of resistance profiles against different antibiotics. (**A**). Frequency of resistance against β-lactam antibiotics. (**B**). Resistance profile against three individual β-lactam antibiotics. MEM: meropenem; AMP: ampicillin and CFM: cefixime. (**C**). Resistance profiles against multiple antibiotic classes. The percentage of isolates that were resistant to an exact and particular number of antimicrobial classes, such as exactly two classes, three classes, four classes of antibiotics, etc., is shown in this table.

In our study, we have investigated the resistance pattern of three different β-lactam antibiotics—ampicillin, meropenem, and cefixime. Of 68 isolates, 11.76% were effectively inhibited by all three β-lactam antibiotics, while 29.41% of isolates developed resistance to all of these antibiotics. Some 39.71% of the isolates could effectively evade at least one β-lactam antibiotic (Figure 5A). Among these antimicrobials, meropenem showed the highest sensitivity, and 32.35% of bacterial isolates rendered meropenem ineffective; this percentage is formidably higher for ampicillin, at 83.82%. Cefixime (CFM), a third-generation cephalosporin derivative, was ineffective against 50% of the isolates (Figure 5B).

### 3.6. Multidrug Resistance Profile

The findings from the investigation of hospital wastewater samples reveal an alarming prevalence of multidrug-resistant isolates (MDR). Specifically, a staggering 86.76% of the isolates were found to exhibit resistance against three or more classes of antibiotics, thereby classifying them as MDR. Moreover, the remaining 13.24% of the isolates were identified to be resistant to two different classes of antibiotics, while none of the isolates demonstrated sensitivity to all seven antimicrobial classes (Figure 5C). In terms of the number of different antibiotic classes to which the isolates exhibited resistance, 5.88%, 41.18%, 26.47%, and 11.76% of the isolates demonstrated resistance against exactly three, four, five, and six different classes of antibiotics, respectively. Alarmingly, one isolate (1.47% of the sample) was found to have developed resistance against all seven classes of antibiotics, which could potentially classify it as a pandrug-resistant (PDR) candidate. Given the gravity of this finding, further investigation is warranted to better understand the implications of this particular isolate.

### 3.7. Prevalence of ESBL Genes

**Figure 6:**
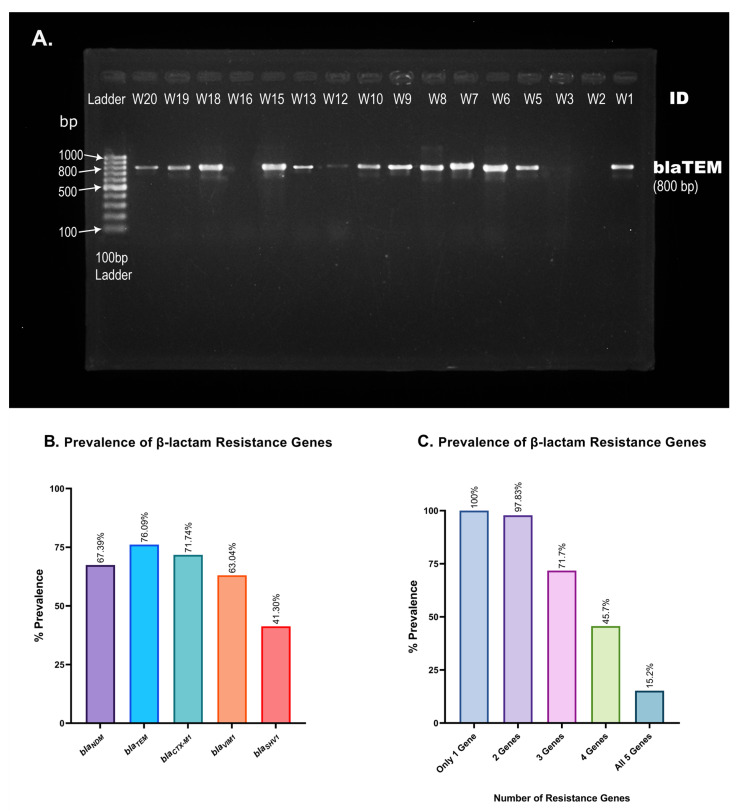
Prevalence of β-lactam resistance genes in hospital wastewater isolates. (**A**). Representative picture of *bla_TEM_* gene visualization. PCR amplification of *bla_TEM_* produces an 800 base-pair long PCR amplicon. A 100 bp gene ladder was used to measure the size of PCR products obtained from different isolates (W1, W2, …, W20). (**B**). Percent prevalence of each of the five resistance genes screened using conventional PCR and gel electrophoresis. *bla_TEM_* and *bla_SHV1_* were found in the most and least isolates, respectively. (**C**). Presence of multiple β-lactam resistance genes in a single isolate. The number of isolates carrying multiple β-lactam resistance genes is alarmingly high, as shown in the bar chart.

ESBL genes encode β-lactamase, which equips bacteria with the ability to hydrolyze antibiotics of β-lactam class. In this study, we evaluated the prevalence of five ESBL genes in hospital wastewater isolates. PCR amplification of ESBL genes and gel electrophoresis (Figure 6A) of 46 of the MDR isolates reveal the widespread prevalence of *bla_TEM_* and *bla_CTX-M1_*, at 76.09% and 71.74% prevalence, respectively, immediately followed by *bla_VIM1_*, with 63.04% prevalence (Table 4). *bla_SHV1_* appeared with a frequency of 41.30%, and despite being a relatively newly identified ESBL gene [32], *bla_NDM_* has already been incorporated into 67.39% of the isolates (Table 4 and Figure 6B). Some 15.2% of the wastewater isolates carried all five of the β-lactam genes that we have analyzed, and 97.83% carried at least two resistance genes (Figure 6C).

### 3.8. Mutational Analysis of Quinolone Resistance-Determining Regions

**Figure 7:**
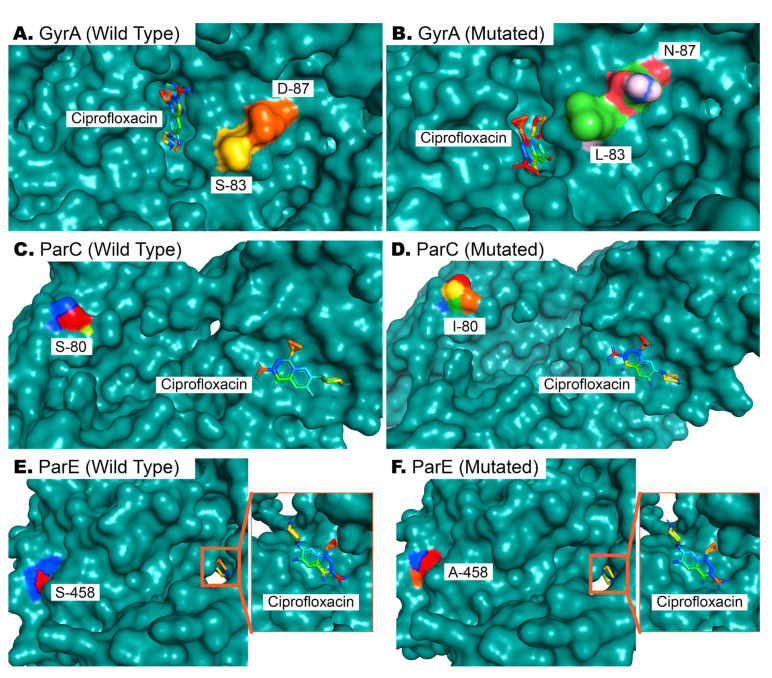
Docking of ciprofloxacin with wild-type and mutated GyrA, ParC, and ParE proteins. (**A**,**B**). Wild-type and mutated bacterial GyrA proteins, wherein two mutations in QRDRs affect the binding of ciprofloxacin to its binding site. (**C**,**D**). The 80th residue of ParC is serine in wild-type ParE proteins, and its mutation to isoleucine does not affect drug binding but reduces affinity. (**E**,**F**). As in (**C**,**D**), the drug binding pose is not affected by the S458A mutation in ParE, but results in reduced affinity of ParE for ciprofloxacin (Table 5).

A preliminary screening was performed on MDR isolates exhibiting resistance to ciprofloxacin and possessing target genes within their genome. Two *E. coli* and two *Klebsiella* spp. isolates were then randomly selected for sequencing and mutation analysis to gain insight into the mutational underpinnings of ciprofloxacin resistance. Five missense mutations were observed in the QRDRs of *gyrA*, *parC*, and *parE* genes. Variants S83L and D87N of *gyrA*, S80I of *parC*, and S458A and I529L of *parE* were present in all (*n* = 4) of the isolates. Mutations in the QRDRs of *gyrA* lead to an altered position of ciprofloxacin binding to the target site of GyrA (Figure 7A,B). This mutation further decreases the affinity of antibiotics to the target site from an average of −5.11 kcal/mol to −5.02 kcal/mol. A detailed analysis of mutation-wise affinity changes is provided in Table 5. Mutation in ParC (S80I) does not affect the binding conformation of ciprofloxacin to ParC (Figure 7C,D), but decreases the affinity slightly (Table 5). Mutations in ParE (S458A and I529L) also do not affect the conformation of ciprofloxacin binding (Figure 7E,F), since these QRDRs are not proximal to drug binding sites, as observed via docking analysis. However, ligand–protein interaction analysis with AutoDock4 [29] revealed a significant decrease in the target site’s affinity for ciprofloxacin in the latter case (Table 5).

One missense mutation never previously identified in Bangladesh, I529L of *parE,* was investigated thoroughly to determine its effect on ciprofloxacin binding affinity. An I529L missense mutation in addition to S458A in *parE* increases the available free energy (ΔG) from −6.99 kcal/mol to −6.39 kcal/mol in its optimal binding position, leading to a reduced affinity for ciprofloxacin and subsequently compromised drug efficacy.

## 4. Discussion

The attempt to create an evidence-based comparative resistance profile of hospital wastewater is a new undertaking in our country. This resistance profile is a good indicator to delineate how fast microbial resistance is spreading into the environment around hospitals. However, it is not only the unsafe disposal of hospital waste that is responsible for turning this city into an antimicrobial resistance hotspot; the easy accessibility of over-the-counter antibiotics in the hospital area of Dhaka, which was previously reported by Rousham et al., is also responsible [33]. Additionally, as untreated wastewater might include significant amounts of bacteria that are resistant to antibiotics and antibiotic residues, insufficient wastewater treatment facilities in hospital areas may facilitate the spread of AMR and MDR.

Hospital wastewater is considered a crucial source of the spread of antimicrobial resistance around the globe. The way in which the discharge of wastewater originating from healthcare facilities, including hospital wastewater, remarkably augments the concentrations of antibiotic-resistant bacteria has been extensively reviewed [34,35]. In a study conducted on hospital wastewater collected from a COVID-19-specific hospital in Saudi Arabia, Wang et al. raised issues regarding the spread of antimicrobial resistance (AMR) during the COVID-19 pandemic, which may have resulted from the usage of antibiotics and untreated hospital wastewater [36]. In China, antibiotics like cefradine, cefepime, and ofloxacin were detected in alarming concentrations and frequencies in hospital wastewater, and bacterial isolates collected from those sources had an alarmingly high prevalence of antibiotic resistance genes like *bla_GES-1_, qnrA, bla_OXA-1_, bla_OXA-10_* and *bla_TEM-1_* [37].

Results from our study show that several of the tested antibiotics had little to no effect on the bacterial isolates from hospital wastewater. Given the potential for the growth of bacteria that are resistant to antibiotics in wastewater, which can result in the development and spread of multidrug-resistant strains, this is especially problematic. Indeed, the majority of the bacterial isolates were found to be multidrug-resistant according to our findings, indicating that MDR is a serious issue in hospital areas.

**Figure 8:**
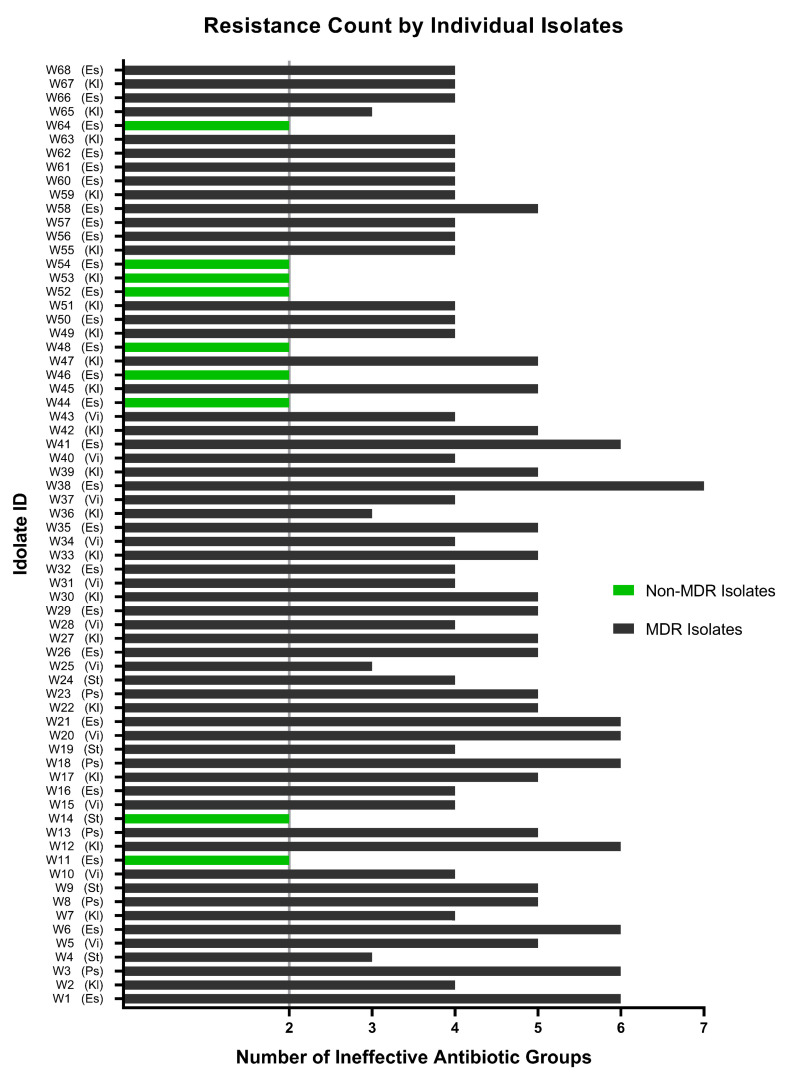
Resistance profile of each of the isolates exposed to all eleven antibiotics of seven classes. A total of 9 isolates (indicated by green bars) have been found to be non-MDR, and the remaining 59 wastewater isolates were marked as MDR (indicated by black bars) to a varying extent, ranging from resistance to three to seven classes of antibiotics. Es: *Escherichia coli*; Kl: *Klebsiella* spp.; Ps: *Pseudomonas aeruginosa*; St: *Staphylococcus aureus*; Vi: *Vibrio cholerae*.

None of the 68 isolates collected from untreated hospital wastewater have shown resistance to less than two classes of antibiotics (Figure 5C and Figure 8), which is quite frustrating, as physicians need to have a thorough antibiogram of an individual before prescribing any antibiotic medication. Some of the isolates had developed extensively drug-resistant (XDR) traits (being resistant to five or more of the seven classes), and one possible pandrug-resistant (PDR) trait was also observed. However, these results are not conclusive, as XDR and PDR are difficult to determine, especially when using a limited selection of antibiotics.

The excessive use of antibiotics in hospitals, lands, and animal farms is the major source of antibiotic-induced bacterial resistance. Over half of hospitalized patients are prescribed broad-spectrum antibiotics such as β-lactams, according to Fridkin et al., and around a third of those prescriptions are unnecessary or inappropriate [38]. These antibiotics end up in adjacent sewage systems and frequently end up in water bodies, leading to an increase in microbial community resistance. A recent study in Bangladesh indicated an alarming rate of contamination of foods and vegetables by multidrug-resistant isolates, which can be exacerbated by hospital wastewater gaining access to the municipal sewage system [39]. Being more cautious when prescribing antibiotics and following appropriate guidelines have the potential to lessen, if not completely prevent, this calamity.

*E. coli* in our study showed almost absolute resistance against erythromycin, ampicillin, and azithromycin (Figure 4B), all of which are still some of the most widely prescribed antibiotics in Bangladesh. These findings align with previous reports that noted the higher resistance of *E. coli* to these antibiotics [40]. Colistin, an 80-year-old antibiotic, could still inhibit most of the isolates of hospital wastewater, except for *Vibrio cholerae*. The prevalence of colistin-resistant *Vibrio cholerae* has not previously been reported and this might be a new threat to public health [40]. Antibiotic use, especially that of azithromycin, surged in Bangladesh during the COVID-19 pandemic, worsening the country’s already out-of-control over-the-counter antibiotic sales. Some 3.5% of those who took antibiotics without a doctor’s prescription during the pandemic were resistant to those drugs [41]. Apart from the hospital wastewater, excessive use of antibiotics in Bangladesh has previously been reported; these antibiotics are mostly unprescribed, and end up mixing with community sewage water [42]. The use of antibiotics during the COVID-19 pandemic may have contributed to a higher occurrence of multidrug-resistant bacteria in hospital wastewater, but we are unable to draw that conclusion from our data without further systemic research.

β-lactam resistance genes appeared in a higher percentage of isolates than in many previous studies, specially *bla_NDM_* and *bla_CTX-M1_* [43,44]. The *bla_CTX m_* group of genes among the multidrug-resistant *E. coli* were previously found to be associated with extraintestinal infections in Bangladesh [44]. Some 71.74% of the MDR isolates in our study expressed *bla_CTX-M1_*, a member of the extended-spectrum beta-lactamase gene, which poses a serious threat if hospital waste disposal is not regulated and hospital sewage water is not decontaminated properly.

Multidrug-resistant bacteria are not just the product of horizontal gene transfer, but also of antibiotic-induced mutagenesis [45]. Antibiotics and antibiotic residues in wastewater cause stress that leads to altered expression of crucial genes like *tetA* and *tetB,* and mutation in genes like *gyrA*, *parC*, or *parE*, which eventually results in fluoroquinolone resistance. A new mutation in *parE* (I152L) was identified in our study, which is novel in Bangladesh and poorly documented internationally; this might be a result of such stress caused by antibiotic residues. Although this QRDR is distal to the ciprofloxacin-binding site, it affects protein–antibiotic interaction and synergistically affects antimicrobial efficacy when additional mutation accumulates. Ampicillin was found to be least potent against wastewater isolates, meaning that the use of this antibiotic will less likely benefit patients, but might stress bacteria, which might lead to additional resistance against additional β-lactam antibiotics. From our analysis, we have noticed a reduced affinity of ParE to ciprofloxacin (Table 5), which may lead to increased resistance against fluoroquinolones if the mutated gene is propagated on a large scale. We must interpret this mutation as a warning to stop using unprescribed or empiric antimicrobial therapy, and to dispose of hospital waste carefully.

The association of mutations in fluoroquinolone resistance genes with ciprofloxacin resistance is supported by many prior studies [46,47]. A recent study by Z. Mahmud et al. with *E. coli* isolates from diarrhea patients revealed a reduced affinity of GyrA for ciprofloxacin due to S83L and D87N missense mutation [30]. Molecular docking simulation in our study replicates previous findings, as in our observation, the combined effect of these two mutations affects the most optimal binding of ciprofloxacin (Figure 7A,B). These mutations are already quite widespread, but mutations like S80I of ParC and S458A and I529L of ParE are not. The latter mutations have limiting effects on ciprofloxacin’s effects on bacteria, as observed from our docking analysis (Table 5).

Modifications in the amino acid sequence within the quinolone resistance-determining regions of these genes have been found to bring about altered binding affinity and intermolecular interaction between proteins and fluoroquinolones. The antibiotics belonging to this class are specifically designed to target a particular site, and any modification in the binding affinity or target site conformation invariably leads to either binding failure or inefficient affinity. Unless hospital wastewater is treated or purified before it is allowed to merge with community drainages, particular resistant isolates may easily gain access to foods, agricultural lands, and households.

Multidrug resistance in hospital wastewater was previously observed to be higher than in community wastewater, and by a significant margin [48]; since in Dhaka city, untreated hospital wastewater merges with community wastewater (Figure 1 and Figure 2), people residing closer to sewage drainage sites are at risk of being infected with MDR bacteria originating from hospitals.

As our research is restricted to only five bacterial species, the information and analysis presented in this article do not include the complete resistance patterns and prevalence of all the resistant isolates found in hospital wastewater. Due to their abundance in wastewater [49], certain species were specifically chosen. That being said, it is probable that many other bacterial species demonstrate comparable or even more substantial levels of resistance to antibiotics; nonetheless, this study provides a broad picture of the resistance patterns in wastewater from the aforementioned hospitals.

By reducing the overuse and abuse of antibiotics in the hospital context, improved antibiotic stewardship policies can help to slow the emergence and spread of bacteria that are resistant to antibiotics. Investments in infrastructure for wastewater treatment can also aid in halting the development of antibiotic-resistant microorganisms in wastewater and the environment. We can protect public health and work to stop the emergence and spread of germs that are resistant to antibiotics by putting effective actions into place. To address this crucial public health issue, further research is required to better understand the causes of antibiotic resistance in hospital wastewater, and to identify appropriate therapies.

## Figures and Tables

**Table 1:** Antibiotic discs of seven antibiotic classes used in the current study.

Antibiotic Class	Antibiotic Name	Acronym	Disc Conc.
Macrolides	Azithromycin	AZM	15 µg
	Erythromycin	E	5 µg
β-lactam antibiotics	Ampicillin	AMP	10 µg
	Meropenem	MEM	10 µg
	Cefixime	CFM	5 µg
Protein synthesis inhibitors	Tetracycline	TE	30 µg
	Chloramphenicol	C	30 µg
Glycopeptide	Vancomycin	VA	30 µg
Polymyxin	Colistin	CT	10 µg
Sulfonamide	Sulfamethoxazole-trimethoprim	SXT	25 µg
Fluoroquinolone	Ciprofloxacin	CIP	5 µg

**Table 2:** Specification of the primers used in the current study.

Gene	Primers (5′→3′)	Tm	Amplicon	Reference
*bla_NDM_*-F	GGTTTGGCGATCTGGTTTTC	52 °C	621 bp	[22]
*bla_NDM_*-R	CGGAATGGCTCATCACGATC			
*bla_TEM_*-F	CATTTCCGTGTCGCCCTTATTC	58 °C	800 bp	[23]
*bla_TEM_*-R	CGTTCATCCATAGTTGCCTGAC			
*bla_VIM1_*-F	CCGATGGTGTTTGGTCGCAT	58 °C	391 bp	[24]
*bla_VIM1_*-R	GAATGCGCAGCACCAGGA			
*bla_SHV1_*-F	AGCCGCTTGAGCAAATTAAAC	58 °C	713 bp	[25]
*bla_SHV1_*-R	ATCCCGCAGATAAATCACCAC			
*bla_CTX-M1_*-F	CGTCACGCTGTTGTTAGGAA	55 °C	781 bp	[26]
*bla_CTX-M1_*-R	ACGGCTTTCTGCCTTAGGTT			
*gyrA*-F	AAATCTGCCCGTGTCGTTGGT	60 °C	344 bp	[15]
*gyrA*-R	GCCATACCTACGGCGATACC			
*gyrB*-F	ATGGATAAAGAAGGCTACAGCA	57 °C	617 bp	[15]
*gyrB*-R	TCGACGTCCGCATCGGTCAT			
*parC*-F	CTGAATGCCAGCGCCAAATT	60 °C	188 bp	[15]
*parC*-R	GCGAACGATTTCGGATCGTC			
*parE*-F	GACCGAAAGCTACGTCAACC	60 °C	958 bp	[15]
*parE*-R	GTTCGGATCAAGCGTGGTTT			

**Table 3:** Comparative resistance profiles of the three most prevalent microbial species isolated from hospital wastewater.

Antibiotics	Resistance Prevalence		
	*E. coli*	*Klebsiella* spp.	*Vibrio cholerae*
Colistin	15.69%	4.76%	100%
Erythromycin	96.08%	90.48%	18.18%
Meropenem	19.61%	42.86%	90.91%
Sulfamethoxazole-Trimethoprim	41.18%	42.86%	9.09%
Tetracycline	56.86%	71.43%	18.18%
Ampicillin	82.35%	90.48%	100%
Azithromycin	90.20%	90.48%	18.18%
Cefixime	47.06%	42.86%	100%
Chloramphenicol	37.25%	52.38%	100%
Vancomycin	58.82%	52.38%	36.36%
Ciprofloxacin	62.75%	66.67%	54.55%

**Table 4:** Prevalence of five β-lactam resistance genes in hospital wastewater isolates.

Gene	Percent Prevalence
*bla_NDM_*	67.39%
*bla_TEM_*	76.09%
*bla_CTX-M1_*	71.74%
*bla_VIM1_*	63.04%
*bla_SHV1_*	41.30%

**Table 5:** Effects of mutations in the QRDRs of quinolone resistance genes on antibiotic–protein binding affinity.

	Affinity (ΔG, kcal/mol)					
	GyrA		ParC		ParE	
Poses	Wild	Mutant (S83L, D87N)	Wild	Mutant (S80I)	Wild	Mutant (S458A, I529L)
1	−5.27	−5.26	−5.57	−5.56	−6.99	−6.39
2	−5.22	−5.25	−5.51	−5.55	−6.90	−6.38
3	−5.21	−4.96	−5.46	−5.51	−6.75	−6.37
4	−5.20	−4.95	−5.43	−4.94	−6.32	−6.36
5	−5.20	−4.94	−5.07	−4.89	−6.31	−6.30
6	−5.18	−4.93	−4.94	−4.84	−6.30	−6.29
7	−4.83	−4.93	−4.93	−4.84	−6.28	−6.05
8	−4.80	−4.92	−4.47	−4.40	−6.25	−5.93
Average	−5.11	−5.02	−5.17	−5.07	−6.51	−6.26

## Data Availability

The datasets that originated in this study will be available upon request.
